# Supplementary material for: Publicly funded interfacility ambulance transfers for surgical and obstetrical conditions: A cross sectional analysis in an urban middle-income country setting
Source: PLoS One. 2020 Nov 6;15(11):e0241553. doi: 10.1371/journal.pone.0241553 (PMC7647066; doi:10.1371/journal.pone.0241553)
Supplement: Attachment — Submitted filename: PONE-D-20-00032.pdf [file pone.0241553.s001.pdf]

PLOS ONE

Publicly Funded Interfacility Ambulance Transfers for Surgical and Obstetrical Conditions: A Cross Sectional Analysis in the Urban Middle-Income Country Setting

--Manuscript Draft--

**Manuscript Number:**	PONE-D-20-00032
**Article Type:**	Research Article
**Full Title:**	Publicly Funded Interfacility Ambulance Transfers for Surgical and Obstetrical Conditions: A Cross Sectional Analysis in the Urban Middle-Income Country Setting
**Short Title:**	Interfacility ambulance transfers in middle-income country
**Corresponding Author:**	Rachel E NeMoyer, MD, MPH<br>Rutgers Robert Wood Johnson Medical School New Brunswick<br>New Brunswick, NJ UNITED STATES
**Keywords:**	middle-income countries, interfacility ambulance transfers, global health
**Abstract:**	Introduction : Interfacility transfers may reflect a time delay for definitive surgical care, but few studies have examined the prevalence of interfacility transfers in the urban LMIC setting. The aim of this study is to determine the number of interfacility transfers due to surgical and obstetric conditions in an urban MIC setting to better understand access to definitive surgical care among LMIC patients.<br>Methods : A retrospective analysis of public interfacility transfer records was conducted from April 2015 to April 2016 in Cali, Colombia. Data were obtained from the single municipal ambulance agency providing publicly funded ambulance transfers for the city. Interfacility transfers were defined as any patient transfer between two healthcare facilities. Descriptive statistics were calculated to compare the number of interfacility transfers among public vs private healthcare facilities and diagnostic condition. Logistic regression was used to compare triage level (severity) of transfer and facility type (public vs. private).<br>Results : 31,659 patient transfers occurred over the 13-month study period. 21,790 of all transfers were interfacility transfers. 7,808 (34.6%) patients required interfacility transfer for surgical conditions with an additional 2,244 (10.3%) patients transferred for obstetric conditions. The most common surgical conditions requiring interfacility transfer were fractures (1,227, 5.4%), appendicitis (913, 4.1%), wounds (871, 3.9%), abdominal pain (818, 3.6%), trauma (652, 2.9%), and acute abdomen (271, 1.2%). Surgical conditions accounted for more interfacility transfers proportionally among public vs. private facilities (33% vs. 15%; p <0.001).<br>Conclusion : Surgical and obstetric conditions account for nearly half of all interfacility ambulance transfers. The most common reasons for transfer are basic surgical disease with public healthcare facilities transferring a greater proportion of patient with surgical conditions when compared to private facilities.
**Order of Authors:**	Paul Truche
	Rachel E NeMoyer, MD, MPH
	Sara Patiño-Franco
	Juan P Herrera-Escobar
	Myerlandi Torres
	Luis F Pino
	Gregory L Peck
**Opposed Reviewers:**	
**Additional Information:**	
**Question**	**Response**
**Financial Disclosure**	The funders had no role in study design, data collection and analysis, decision to publish, or preparation of the manuscript.

Powered by Editorial Manager® and ProduXion Manager® from Aries Systems Corporation

Enter a financial disclosure statement that describes the sources of funding for the work included in this submission. Review the submission guidelines for detailed requirements. View published research articles from *PLOS ONE* for specific examples.<br><br>This statement is required for submission and **will appear in the published article** if the submission is accepted. Please make sure it is accurate.<br><br>**Unfunded studies**<br>Enter: *The author(s) received no specific funding for this work.*<br><br>**Funded studies**<br>Enter a statement with the following details:<br>• Initials of the authors who received each award<br>• Grant numbers awarded to each author<br>• The full name of each funder<br>• URL of each funder website<br>• Did the sponsors or funders play any role in the study design, data collection and analysis, decision to publish, or preparation of the manuscript?<br>• **NO** - Include this sentence at the end of your statement: *The funders had no role in study design, data collection and analysis, decision to publish, or preparation of the manuscript.*<br>• **YES** - Specify the role(s) played.<br><br>* typeset	
**Competing Interests**<br><br>Use the instructions below to enter a competing interest statement for this submission. On behalf of all authors, disclose any competing interests that could be perceived to bias this work—acknowledging all financial support and any other relevant financial or non-financial competing interests.<br><br>This statement **will appear in the published article** if the submission is accepted. Please make sure it is accurate. View published research articles from *PLOS ONE* for specific examples.	The authors have declared that no competing interests exist.

Powered by Editorial Manager® and ProduXion Manager® from Aries Systems Corporation

**NO authors have competing interests**<br><br>Enter: *The authors have declared that no competing interests exist.*<br><br>**Authors with competing interests**<br><br>Enter competing interest details beginning with this statement:<br><br>*I have read the journal's policy and the authors of this manuscript have the following competing interests: [insert competing interests here]*<br><br>* typeset	
**Ethics Statement**<br><br>Enter an ethics statement for this submission. This statement is required if the study involved:<br><br>• Human participants<br>• Human specimens or tissue<br>• Vertebrate animals or cephalopods<br>• Vertebrate embryos or tissues<br>• Field research<br><br>Write "N/A" if the submission does not require an ethics statement.<br><br>General guidance is provided below. Consult the submission guidelines for detailed instructions. **Make sure that all information entered here is included in the Methods section of the manuscript.**	n/a

Powered by Editorial Manager® and ProduXion Manager® from Aries Systems Corporation

**Format for specific study types**<br><br>**Human Subject Research (involving human participants and/or tissue)**<br>• Give the name of the institutional review board or ethics committee that approved the study<br>• Include the approval number and/or a statement indicating approval of this research<br>• Indicate the form of consent obtained (written/oral) or the reason that consent was not obtained (e.g. the data were analyzed anonymously)<br><br>**Animal Research (involving vertebrate animals, embryos or tissues)**<br>• Provide the name of the Institutional Animal Care and Use Committee (IACUC) or other relevant ethics board that reviewed the study protocol, and indicate whether they approved this research or granted a formal waiver of ethical approval<br>• Include an approval number if one was obtained<br>• If the study involved *non-human primates*, add *additional details* about animal welfare and steps taken to ameliorate suffering<br>• If anesthesia, euthanasia, or any kind of animal sacrifice is part of the study, include briefly which substances and/or methods were applied<br><br>**Field Research**<br><br>Include the following details if this study involves the collection of plant, animal, or other materials from a natural setting:<br>• Field permit number<br>• Name of the institution or relevant body that granted permission	
**Data Availability**<br><br>Authors are required to make all data underlying the findings described fully available, without restriction, and from the time of publication. PLOS allows rare exceptions to address legal and ethical concerns. See the PLOS Data Policy and FAQ for detailed information.	Yes - all data are fully available without restriction

Powered by Editorial Manager® and ProduXion Manager® from Aries Systems Corporation

A Data Availability Statement describing where the data can be found is required at submission. Your answers to this question constitute the Data Availability Statement and **will be published in the article**, if accepted.<br><br>**Important:** Stating 'data available on request from the author' is not sufficient. If your data are only available upon request, select 'No' for the first question and explain your exceptional situation in the text box.<br><br>Do the authors confirm that all data underlying the findings described in their manuscript are fully available without restriction?	
**Describe where the data may be found in full sentences. If you are copying our sample text, replace any instances of XXX with the appropriate details.**<br><br>• If the data are **held or will be held in a public repository**, include URLs, accession numbers or DOIs. If this information will only be available after acceptance, indicate this by ticking the box below. For example: *All XXX files are available from the XXX database (accession number(s) XXX, XXX.*).<br>• If the data are all contained **within the manuscript and/or Supporting Information files**, enter the following: *All relevant data are within the manuscript and its Supporting Information files.*<br>• If neither of these applies but you are able to provide **details of access elsewhere**, with or without limitations, please do so. For example:<br><br>*Data cannot be shared publicly because of [XXX]. Data are available from the XXX Institutional Data Access / Ethics Committee (contact via XXX) for researchers who meet the criteria for access to confidential data.*<br><br>*The data underlying the results presented in the study are available from (include the name of the third party*	All relevant data are within the manuscript and its Supporting Information files.

and contact information or URL).<br>• This text is appropriate if the data are owned by a third party and authors do not have permission to share the data.<br><br>* typeset	
Additional data availability information:	

Powered by Editorial Manager® and ProduXion Manager® from Aries Systems Corporation

Dear Editor,

We are excited to share our manuscript *entitled Publicly Funded Interfacility Ambulance Transfers for Surgical and Obstetrical Conditions: A Cross Sectional Analysis in the Urban Middle-Income Country Setting* which my coauthors Paul Truche, Rachel E. NeMoyer, Sara Patiño-Franco, Juan P. Herrera-Escobar, Myerlandi Torres, Luis F. Pino and I wish to submit for publication in PLOS One.

This work represents an international collaboration between academic partners in the United States and Colombia as well as government sector involvement to evaluate the role and scope of surgical disease among interfacility ambulance transfers in the middle-income country setting. The manuscript is based on an analysis of primary data from the publicly funded ambulance system, Salud Centro, with the aim to determine the number of interfacility transfers due to surgical and obstetric conditions in an urban MIC setting to better understand access to definitive surgical care among MIC patients.

The manuscript sheds light on the systems levels strain that untreated surgical disease has on the public health system and contextualizes this within Colombia's healthcare service framework. Our work fits within the timeliness domain outlined by the Lancet Commission on Global Surgery as we investigate systems level delays in surgical healthcare access in the MIC setting. Ultimately, we have shown that surgical conditions make up a significant proportion of all interfacility ambulance transfers in Cali Colombia suggesting that even in the urban middle-income country setting, there is need for policy implementation towards improving access to timely and definitive surgical healthcare for the population. We believe this manuscript aligns with the aims of PLOS One and will be of benefit to surgeons working in the measurement of the public healthcare sector in low- and middle-income countries across the world.

This manuscript represents original work and has not been published or under consideration for publication in any other journals. We look forward to the opportunity to publish our results and further inform the field of academic global surgery and surgical system science.

Sincerely,

Gregory L. Peck, DO
Department of Surgery, Rutgers Robert Wood Johnson Medical School
Department of Health Systems and Policy, Rutgers School of Public Health

Cover Letter

Click here to download Manuscript Publicly Funded Interfacility .docx

Publicly Funded Interfacility Ambulance Transfers for Surgical and Obstetrical Conditions: A Cross Sectional Analysis in the Urban Middle-Income Country Setting

Paul Truche MD; Rachel E. NeMoyer MD, MPH; Sara Patiño-Franco MD; Juan P. Herrera-Escobar MD, MPH; Myerlandi Torres; Luis F. Pino MD; Gregory L. Peck DO

Paul Truche: Department of Surgery, Rutgers Robert Wood Johnson Medical School; ptruche@hsph.harvard.edu

Rachel E. NeMoyer MD, MPH: Department of Surgery, Rutgers Robert Wood Johnson Medical School; ren32@rwjms.rutgers.edu

Sara Patiño-Franco MD: Universidad de Antioquia, Colombia. sarapatinofranco@gmail.com

Juan P. Herrera-Escobar MD, MPH: Center for Surgery and Public Health, Brigham and Women’s Hospital; jherrera@hsph.harvard.edu

Myerlandi Torres: Red de salud del Centro, Pool ambulancias públicas, Cali, Colombia. miyertorres@hotmail.com

Luis F. Pino MD: Department of Surgery, Universidad Del Valle, Colombia; luis.pino@correounivalle.edu.co

Gregory L. Peck DO, FACS: Department of Surgery, Rutgers Robert Wood Johnson Medical School, Department of Health Systems and Policy, Rutgers School of Public Health; peckgr@rwjms.rutgers.edu

write the acronym out in full
Idem for LMIC

Introduction: Interfacility transfers may reflect a time delay for definitive surgical care, but few studies have examined the prevalence of interfacility transfers in the urban LMIC setting. The aim of this study is to determine the number of interfacility transfers due to surgical and obstetric conditions in an urban MIC setting to better understand access to definitive surgical care among LMIC patients.

Methods: A retrospective analysis of public interfacility transfer records was conducted from April 2015 to April 2016 in Cali, Colombia. Data were obtained from the single municipal ambulance agency providing publicly funded ambulance transfers for the city. Interfacility transfers were defined as any patient transfer between two healthcare facilities. Descriptive statistics were calculated to compare the number of interfacility transfers among public vs private healthcare facilities and diagnostic condition. Logistic regression was used to compare triage level (severity) of transfer and facility type (public vs. private).

Results: 31,659 patient transfers occurred over the 13-month study period. 21,790 of all transfers were interfacility transfers. 7,808 (34.6%) patients required interfacility transfer for surgical conditions with an additional 2,244 (10.3%) patients transferred for obstetric conditions. The most common surgical conditions requiring interfacility transfer were fractures (1,227, 5.4%), appendicitis (913, 4.1%), wounds (871, 3.9%), abdominal pain (818, 3.6%), trauma (652, 2.9%), and acute abdomen (271, 1.2%). Surgical conditions accounted for more interfacility transfers proportionally among public vs. private facilities (33% vs. 15%; p <0.001).

Conclusion: Surgical and obstetric conditions account for nearly half of all interfacility ambulance transfers. The most common reasons for transfer are basic surgical disease with public healthcare facilities transferring a greater proportion of patient with surgical conditions when compared to private facilities.

Introduction:

Emergency and essential surgical conditions account for up to one third of the total global burden of disease.[1] Treatment of these surgical conditions requires delivery of both timely and definitive surgical care that relies heavily on prehospital triage systems. Prehospital triage systems have proven difficult to systematically examine in low and middle income countries (LMICs).[2] Given this known challenge, the World Health Organization and the World Bank advocate for the collection, analysis, and interpretation of data that informs a timely healthcare delivery integral to the achievement of definitive surgical care globally.[3–5]

In 2015, the Lancet Commission on [illegible] a target for 80% of the population in low and middle income countries (LMICs) to have timely access to surgical care within 2 hours by 2030.[6] The number of patients who reach a facility within 2 hours, but still do not receive definitive surgical care is not well quantified in the LMIC setting. A "Three Delay" framework consisting of 1) delays in deciding to seek care at a facility 2) delays in reaching a facility and 3) delays in the delivery of definitive care once at a facility has been proposed in maternal-fetal health literature to assess the continuum of delays in low resource settings.[7,8] A thorough understanding of interfacility transfers for surgical conditions can help to identify 2nd and third delays in access to definitive surgical care in LMICs.

LMICs population

In Latin America, where the majority of countries are middle in[illegible]pital trauma triage provides examples of how prehospital care may help [illegible] delays and favorably impact outcomes once definitive care is obtained.[9,10] Prehospital triage systems here are dependent on the development and implementation of health system building blocks including those advocated for within National surgical, obstetric and anesthesia plans (NSOAP).[11] Interfacility transfer, a process within prehospital triage systems, has demonstrated to improve surgical outcomes in low resource regions where long transport times exist related to the above health system building blocks such as infrastructure, geography or policy non-adherence.[12–15] In recognition of how difficult it has been for both low and middle income

could you describe in 3 lines the specificity of Colombia?

countries to formally integrate the prehospital systems with hospital level healthcare delivery and outcomes, action has been called for and important progress has been made with respect to system strengthening.[10,16,17] Access to surgical care in LMICs remains a multi-disciplinary and multi-agency agenda needing further development and implementation.[17–20]

With continued emphasis on surgical system strengthening in all LMICs, evaluation of the nuance between timely arrival and delivery of definitive surgical care is warranted. Interfacility transfers represent a proxy for measuring a distinct aspect of the second and third delay whereby patients reach a facility but must be transferred to obtain the definitive surgical care they need. The aim of this study is to determine the number of interfacility transfers due to surgical and obstetric conditions in an urban MIC setting to better understand access to definitive surgical care among LMIC patients.

Methods

A large retrospective review of ambulance records from April 2015 through April 2016 in Cali, Colombia was performed. Administrative transfer records of Cali's single public ambulance agency, Salud Centro, which provides the majority of both emergency 911 transports and interfacility transfers was analyzed. Variables extracted included patient level data such as: the date of transport, gender, age, origin (primary) facility, destination (secondary) facility, International Classification of Diseases ninth revision (ICD9), transport priority level (urgent, emergent, code blue), and provider specialty receiving transfer.

Subjects included any patient who underwent an interfacility transfer during the study period. Individual facilities included all hospitals and clinics and were categorized as public or private based on review of each facilities website or online listing. Transfers were considered interfacility if both the origin and destination were a hospital or clinic. The primary outcome evaluated was interfacility transfer for a surgical condition. 911 calls including all transports initiated from a home or private physician's office, and discharges including transports with a final destination of a home, were excluded.

Provider specialty accepting transfer was categorized as emergency medicine, surgery, obstetrics, intensive care, internal medicine, pediatrics, or other. Specialties that were considered surgical included ear-nose-and-throat (ENT), cardiothoracic (CT), gastroenterological (GI), general surgery, urology, vascular, surgical oncology, orthopedic surgery, plastic surgery, neurosurgery, ophthalmology, and pediatric surgery.

There were 2,273 unique ICD9 codes in total for the sample population. Individual ICD9 codes categorized as surgical, obstetric, or non-surgical were then subcategorized by condition into one common condition for statistical analysis (i.e., all fracture codes were grouped as fracture). Surgical conditions were identified as conditions that require care rendered by a surgeon. Obstetric conditions included all diagnoses related to pregnancy or childbirth and the related complications thereof.

Descriptive statistics were performed for the number of interfacility transfers and ICD9 diagnoses originating and concluding at a public and/or private hospital (facility type) and patients undergoing interfacility transfer, respectively. Chi squared tests were used to compare categorical variables, and t-tests to compare continuous variables. Chi squared tests were also performed to evaluate the difference in proportion of surgical diagnoses between public and private origin facilities and odds ratios (OR) were calculated. Logistic regression was used to compare triage level to origin facility type. Statistical analyses were performed using the SAS statistical software package version 9.4.

Institutional Review Board (IRB) approval for this study was [illegible]gers Robert Wood Johnson Medical School and the Universidad de Valle with a formal data sharing agreement with Salud Centro Ambulance service. Involved members of respective institutions have representative authorship.

if you had an agreement number?

Results

A total of 31,659 patients were transported by the public ambulance system over the study period. Of the total transports, 21,790 were interfacility transfers originating from one of 425 unique facilities. Fifty of the 425 facilities (12%) contributed to 90% of the burden of transfers, and the top 20 facilities (5%) contributed to 70% of the total transfer burden. Surgical conditions accounted for 34.6% (7,808) of interfacility transfers, obstetric and gynecologic conditions, 10.3%, and nonsurgical conditions, the remaining 55.5%. The proportion of transfers for surgical conditions were higher for public facilities than private facilities (33% vs. 15%; p <0.001).

17,675 patients were transferred from public facilities and 4,580 patients were transferred from private facilities. Public facilities transferred to public facilities 61% of the time, while private facilities transferred to private facilities 77% of the time. These differences were statistically significant (p<0.0001) [Table 1]. By specialty, 6,616 patients were transferred to a surgeon and represented 30.1% of all interfacility transfers. 6,000 patients (27.3%) were transferred to internists, 2,805 patients (12.7%) were transferred to obstetricians, 2,026 patients (9.2%) were transferred to pediatricians, 1,368 patients (6.5%) were transferred to an ICU provider, and 3,104 (14.2%) patients were transferred to other specialties [Table 2].

Table 1

It would be logical to indicate in Tables 1 and 2 whether or not there is a significant difference

**Interfacility Transfers**				
	**All Transfers (n=22,255)**		**Transfers for Surgical/Obstetric Conditions (n=9,987)**	
**Origin Hospital Type**	**Private n (%)**	**Public n (%)**	**Private n (%)**	**Public n (%)**
**Number of Transfers**	4580 (21)	17675 (79)	1466 (15)	8521 (85)
**Destination Hospital**				

Private	3511 (77)	6676 (40)	1025 (70)	2610 (23)
Public	1011 (13)	10592 (60)	417 (30)	5730 (67)
**Gender**				
Male	2498 (55)	9160 (52)	760 (52)	4346 (51)
Female	2082 (45)	8515 (48)	706 (48)	4175 (49)
**Mean Age (years)**	41.2 (SD* 26.2)	46.5 (SD 26.4)	40.0 (SD 24.9)	34.1 (SD 23.8)

*SD: Standard Deviation

Table 2

Transfer by Condition Type

	**Private n (%)**	**Public n (%)**
**Total**	4580 (21)	17675 (79)
Non-surgical	3114 (68)	9152 (52)
Obstetric	212 (5)	2060 (12)
Surgical	1254 (27)	6461 (37)
**Transfer by Specialty Type**		
Intensive Care	251 (5)	1177 (7)
Internal Medicine	827 (18)	5173 (29)
Obstetrics	270 (6)	2535 (14)
Surgery	707 (15)	5909 (33)
Pediatrics	225 (5)	1801 (10)
Other specialist	2300 (50)	1080 (6)

The majority of interfacility transfers were not emergent and classified as “priority” or “non-urgent” transfers. The use of each triage level as an indicator of disease severity was statistically different between public and private facilities; code blue ($p<0.001$), emergent

(p<0.001), and urgent transfers (p<0.001). Private hospitals had a higher proportion of transfers initiated as code blue and/or emergent [Table 3].

Table 3

Odds of interfacility transfer for public versus private facility based on triage level

**Triage level**	**Public n (%)**	**Private n (%)**	**Odds Ratio (95% CI*)**	**p-value**
Code blue	39 (0.2)	701 (3.2)	(44.4-229.8)	**<0.0001**
Emergent	2056 (9.3)	10124 (45.8)	(51.1-100.5)	**<0.0001**
Priority	574 (2.6)	6087 (27.5)	(17.6-21.9)	0.1190
Urgent	1888 (8.5)	473 (2.1)	(37.1-48.3)	**<0.0001**
Non-urgent	1888 (8.5)	473 (2.1)	reference	

*CI: Confidence Interval
Bolded estimates have a significant Wald Chi-Square p-value, p<0.05

The top 21 conditions requiring interfacility transfers included many surgical diagnoses such as fractures, appendicitis, wounds, abdominal pain, trauma, and labor complications [Table 4]. For patients undergoing interfacility transfer for appendicitis, the odds of the transfer originating from a public facility were 4.4x higher than a private facility (OR=4.4, 95% Confidence Interval (CI) 3.3-5.8, p<0.001). For patients transferred for wounds, the odds were 7.5x higher to have originated from a public facility vs. a private facility (OR=7.5, 95% CI 5.2-10.8, p<0.001]). There were no statistical differences between facility type origin for fractures (p<0.06), abdominal pain (p<0.63), or trauma (p<0.92). [Table 5]

Table 4

Odds of interfacility transfer originating from a public versus private facility by diagnosis (reference is private)

Diagnosis by ICD9 Code	n	Public n (%)	Private n (%)	Odds Ratio (95% CI*)	p-value
Fracture	1217	992 (89.4)	225 (10.6)	1.15 (0.99, 1.34)	0.07
Fever	965	863 (94.2)	102 (5.8)	2.25 (1.83, 2.77)	**<0.0001**
Appendicitis	903	851 (94.2)	52 (5.8)	4.40 (3.32, 5.84)	**<0.0001**
Wound	859	829 (96.5)	30 (3.5)	7.46 (5.18, 10.76)	**<0.0001**
Abdominal Pain	809	648 (80.0)	161 (20.0)	1.04 (0.88, 1.25)	0.66
Unspecified Medical Condition	776	111 (14.3)	665 (85.7)	0.04 (0.03, 0.05)	**<0.0001**
MI**	759	608 (80.1)	151 (19.1)	1.04 (0.87, 1.25)	0.68
Pneumonia	733	609 (83.0)	124 (17.0)	1.28 (1.05, 1.56)	0.01
Cerebrovascular Disease	676	525 (77.6)	151 (22.3)	0.90 (0.74, 1.08)	0.25
Trauma	642	509 (79.3)	133 (20.7)	0.99 (0.82, 1.20)	0.92
Labor complication	587	538 (91.7)	49 (8.3)	2.90 (2.16, 3.90)	**<0.0001**
Abortion	499	470 (94.2)	29 (5.8)	4.29 (2.94, 6.25)	**<0.0001**
Infection	436	346 (79.2)	91 (20.8)	0.98 (0.78, 1.24)	0.9
Hypertension	424	351 (82.8)	73 (17.2)	1.25 (0.97, 1.61)	0.09
Chest Pain	368	324 (88)	44 (12)	1.93 (1.40, 2.08)	**<0.0001**
Seizure	361	309 (85.6)	52 (14.4)	1.55 (1.15, 2.08)	0.0031
GI# bleed	311	257 (82.6)	54 (17.4)	1.24 (0.92, 1.66)	0.18
Labor	311	280 (90)	31 (10)	2.36 (1.63, 3.43)	**<0.0001**
CHF+	309	256 (82.9)	53 (17.1)	1.26 (0.93, 1.69)	0.14
COPD++	304	233 (76.6)	71 (23.4)	0.85 (0.65, 1.11)	0.23
Acute Abdomen	270	239 (88.5)	31 (11.5)	0.49 (0.34-0.73)	0.0001

*CI: Confidence Interval; **MI: Myocardial Infarction; #GI: Gastrointestinal bleed; +CHF: Congestive Heart Failure; ++COPD: Chronic Obstructive Pulmonary Disease

Bolded estimates have a significant Wald Chi-Square p-value, p<0.05

Table 5

Odds of interfacility transfer for public versus private facility for common surgical conditions

**Surgical Condition**	**n (%)**	**Odds Ratio (95% CI*)**	**p-value**
Fracture	1,227 (5.4)	1.15 (0.99, 1.34)	0.07
Appendicitis	913 (4.1)	4.40 (3.32, 5.84)	**<0.0001**
Wound	871 (3.9)	7.46 (5.18, 10.76)	**<0.0001**
Abdominal Pain	818 (3.6)	1.04 (0.88, 1.25)	0.66
Trauma	652 (2.9)	0.99 (0.82, 1.20)	0.92

*CI: Confidence Interval
Bolded estimates have a significant Wald Chi-Square p-value, p<0.05

Discussion

Our study reveals that up to one third of patients undergoing interfacility transfers in an urban MIC setting are being transferred for surgical conditions. Common surgical conditions requiring interfacility transfer from a primary healthcare facility include fractures, appendicitis, wounds, abdominal pain, and trauma. When obstetrical conditions are combined with surgical conditions, nearly half of all interfacility transfers are represented. These data define a previously unrecognized, and significant proportion of interfacility transfers that occur due to definitive surgical healthcare being sought at a second healthcare facility in an urban MIC setting.

A number of studies have utilized time to arrival to the nearest hospital as representative of “timely access” to surgical care in LMICs.[21,22] Our study supports the notion that, for a large proportion of patients, definitive surgical care is not delivered upon “timely access” to a first, initial healthcare facility. Interfacility transfers may not be a cause, but instead representative of either a second (delay in reaching care) or a third (delay in receiving care) dela[illegible] to this dual impact, rates of interfacility transfers could be a useful data point [illegible] definitive access to timely surgery in MICs, where absolute times [illegible]tive surgical intervention data may not exist just yet. Previous work conducted in Pakistan has linked inability to pay to delays in interfacility transfer for surgical patients.[23] Our study details a regional setting in which interfacility transfer may reflect the inability to definitively treat a basic surgical condition despite timely access to the first hospital. Modeling of surgical access predominantly rely on estimates of patients’ ability to reach a hospital, and do not account for the possibility of needing an interfacility transfer for definitive surgical healthcare at a second healthcare facility.[21,22,24,25] Because an undetermined third delay occurs within an interfacility transfer process, models that exclude this large proportion of patients likely overestimates “timely access” to definitive surgical healthcare globally.

> why talk about health systems in Asia? Couldn't you refer to some industrialized countries (UK, ???) or South America

Low income country evidence has shown a survival benefit for trauma and obstetrical patients when interfacility transfer is more readily available.[16,26,27] When the entire spectrum of interfacility transfers are considered, including modifying factors of initial pre-hospital system triage that could potentially circumvent the need for interfacility transfer, or improving the efficiency and effectiveness of interfacility transfers when needed, actual second and third delays can be more precisely assessed and addressed. As evidenced by the absolute number and proportion of patients in this study requiring interfacility transfers for surgical conditions, a large population of patients stand to benefit from these surgical system preparedness and service delivery-based considerations.

The factors of delay in timely and quality surgical care delivery represent tangible targets for evidence-based policy improvement of access to definitive surgical healthcare. Although universal health insurance in Colombia has expanded the coverage of healthcare services, timely and quality delivery of definitive healthcare services is thought to vary among socio-economic groups and the regional rural-urban geographic divide.[28,29] The Organization for Economic Co-operation and Development (OECD) reported that *vertical integration* between providers and the over 99 public and private health insurers in Colombia has contributed to a fragmented healthcare delivery, reduced competition at the provider level, and makes it difficult to allocate sparse human resources equitably and efficiently.[30] This vertical integration, where healthcare delivery entities are entwined with individual insurers, makes an outcomes based surgical healthcare system difficult to achieve and complicates cooperation between competitive and cross-sectoral healthcare facilities and markets. The rate of interfacility transfers from our data may corroborate the concerns engendered by the OECD's statement, as definitive delivery of surgical healthcare requires specialized infrastructure and workforce that is not easily expanded across the public and private hospitals within independent healthcare systems.

In our study both public and private hospitals utilize the public sector Emergency Medical Services (EMS) for interfacility transfers. When considering a common condition such as

appendicitis, our data demonstrates that public hospitals have a higher proportion of interfacility transfers for appendicitis when compared to private. The disproportionate rate of public-private interfacility transfers may reflect the imbalance inherent with vertical integration where private hospitals are equipped to deliver definitive surgical healthcare because of a sustainable infrastructure and workforce. Studies in LMICs are replete with respect to the imbalance in healthcare infrastructure and workforce between public and private sectors and the inequitable impact on defined sub-populations.[31–33]

The fact that the most common conditions being transferred by the public sector in our study were largely surgical may reflect a system that varies in infrastructure and workforce capacity key to delivering both timely and definitive surgical healthcare. Importantly, it is not known whether interfacility transfer is a solution to, or an outcome of the *vertical integration* suggested to be present in Colombia.[30] Further data surrounding the interface between vertically independent healthcare entities is needed to understand how to best utilize the limited surgical workforce and infrastructure for the population's need, especially within competitive markets. Analysis of this data could then direct prehospital and interfacility transfer policies in the middle-income country towards a value-based, outcomes driven delivery of definitive surgical care.

When we consider the private sector and interfacility transfer, surgical conditions requiring transfer by private hospitals were more likely to be of the highest urgency level (code blue). This raises the question of whether private hospitals tend to use the publicly funded ambulance system for their sicker patients. Nevertheless, both public and private sector examples in our study represent a cross-sectoral need for policy development that establishes an equitable delivery of timely and definitive, quality healthcare in this region. Prioritization of equitable delivery of definitive surgical healthcare is especially important given the large population burden herein that requires definitive treatment of emergency and essential surgical and obstetrical conditions.

Limitations

Our study examines only interfacility transfers executed through the public ambulance sector and does not account for transfers provided by the other privately-owned ambulance companies in Cali. The analyses conducted are based exclusively on administrative data that is limited to a single public EMS agency that does not include clinical variables other than hospital-based diagnoses codes. Clinical status of patients undergoing transfer was limited to the triage level initiated by the hospital at the time of transfer. Although ICD9 codes were used in our data analysis, local billing practices have an unknown impact on codes chosen and recorded for individual patients. The above are clear examples of potential measurement and information bias, respectively.

Although some hospitals utilizing the public EMS agency were utilized in our analysis, this study does not account for the private ambulances operated by the individual hospitals. Such a large selection bias requires a collaborative cross-agency study to overcome these limitations of our study design. Finally, this study does not take into account the formal and informal transfer agreements that may exist because of contractual commitments between primary and secondary healthcare facilities, the public EMS agency, and local workforce, governance and finance infrastructures. Therefore [illegible] and confounding variables exist with regard to why the secondary hospitals [illegible]d to accept the volume of interfacility transfers exhibited in this study.

could you identify some recommendations or priorities for the regional vocation in accordance or not with the WHO guidelines or others?

Conclusion

Surgical and obstetric conditions account for over half of all interfacility ambulance transfers in this urban MIC setting. The most common reasons for transfer are basic surgical conditions, with public healthcare facilities requiring a greater proportion of interfacility transfers for surgical conditions. Further research towards the nature of hospital interface through prehospital transfers is needed to understand how to best utilize the limited surgical workforce and infrastructure in middle income century. Nevertheless, a foundation for additional research

that links surgical healthcare outcomes data to interfacility transfer has been established and requires further investigation.

References

1. Bickler SN, Weiser TG, Kassebaum N, et al. Global Burden of Surgical Conditions. In: Debas HT, Donkor P, Gawande A, Jamison DT, Kruk ME, Mock CN, eds. *Essential Surgery: Disease Control Priorities, Third Edition (Volume 1)*. Washington (DC): The International Bank for Reconstruction and Development / The World Bank; 2015. http://www.ncbi.nlm.nih.gov/books/NBK333518/. Accessed March 28, 2019.
2. Suryanto null, Plummer V, Boyle M. EMS Systems in Lower-Middle Income Countries: A Literature Review. *Prehospital Disaster Med*. 2017;32(1):64-70. doi:10.1017/S1049023X1600114X
3. Debas. *Debas, H. T., P. Donkor, A. Gawande, D. T. Jamison, M. E. Kruk, and C. N. Mock, Editors. 2015. Essential Surgery. Disease Control Priorities, Third Edition, Volume 1. Washington, DC: World Bank.*
4. Thind A, Hsia R, Mabweijano J, Hicks ER, Zakariah A, Mock CN. Prehospital and Emergency Care. In: Debas HT, Donkor P, Gawande A, Jamison DT, Kruk ME, Mock CN, eds. *Essential Surgery: Disease Control Priorities, Third Edition (Volume 1)*. Washington (DC): The International Bank for Reconstruction and Development / The World Bank; 2015. http://www.ncbi.nlm.nih.gov/books/NBK333513/. Accessed March 12, 2019.
5. WHO | Applying the lessons of maternal mortality reduction to global emergency health. WHO. https://www.who.int/bulletin/volumes/93/6/14-146571/en/. Accessed March 15, 2019.
6. Meara JG, Leather AJM, Hagander L, et al. Global Surgery 2030: Evidence and solutions for achieving health, welfare, and economic development. *The Lancet*. 2015;386(9993):569-624. doi:10.1016/S0140-6736(15)60160-X
7. Barnes-Josiah D, Myntti C, Augustin A. The “three delays” as a framework for examining maternal mortality in Haiti. *Soc Sci Med 1982*. 1998;46(8):981-993.
8. Mgawadere F, Unkels R, Kazembe A, van den Broek N. Factors associated with maternal mortality in Malawi: Application of the three delays model. *BMC Pregnancy Childbirth*. 2017;17(1):219. doi:10.1186/s12884-017-1406-5
9. Sorensen MJ, Recklinghausen FM von, Fulton G, Burchard KW. Secondary Overtriage: The Burden of Unnecessary Interfacility Transfers in a Rural Trauma System. *JAMA Surg*. 2013;148(8):763-768. doi:10.1001/jamasurg.2013.2132
10. Arreola-Risa C, Mock CN, Lojero-Wheatly L, et al. Low-cost improvements in prehospital trauma care in a Latin American city. *J Trauma*. 2000;48(1):119-124.
11. Albutt K, Citron I, Sonderman K, Nthele M, Bekele A, Maongezi S, et al. National Surgical Obstetric and Anaesthesia Planning: Process and Consensus Recommendations . Dubai, United Arab Emirates: Harvard Medical School Center for Global Health Delivery–Dubai; 2018. 2018-dubai_nsoap_policy_brief.pdf. https://ghd-dubai.hms.harvard.edu/files/ghd_dubai/files/2018-dubai_nsoap_policy_brief.pdf?m=1525866968. Accessed March 29, 2019.
12. Razzak JA, Kellermann AL. Emergency medical care in developing countries: Is it worthwhile? *Bull World Health Organ*. 2002;80(11):900-905.
13. Henry JA, Reingold AL. Prehospital trauma systems reduce mortality in developing countries: A systematic review and meta-analysis. *J Trauma Acute Care Surg*. 2012;73(1):261-268. doi:10.1097/TA.0b013e31824bde1e
14. Callese TE, Richards CT, Shaw P, et al. Trauma system development in low- and middle-income countries: A review. *J Surg Res*. 2015;193(1):300-307. doi:10.1016/j.jss.2014.09.040

15. Sriram V, Gururaj G, Razzak JA, Naseer R, Hyder AA. Comparative analysis of three prehospital emergency medical services organizations in India and Pakistan. *Public Health*. 2016;137:169-175. doi:10.1016/j.puhe.2016.02.022
16. Vera-López JD, Pérez-Núñez R, Gómez-García L, Hidalgo-Solórzano E, Fraga-Sastrías JM. [The Emergency Medical System's response and its association with different outcomes in traffic accident victims in Mexican cities]. *Cad Saude Publica*. 2018;34(10):e00144916. doi:10.1590/0102-311X00144916
17. Peralta LMP. The prehospital emergency care system in Mexico City: A system's performance evaluation. *Prehospital Disaster Med*. 2006;21(2):104-111.
18. Pinet LM. [Prehospital emergency care in Mexico City: The opportunities of the healthcare system]. *Salud Publica Mex*. 2005;47(1):64-71.
19. Mallon W, Valenzuela R, Salway R, Shoenberger J, Swadron S. THE SPECIALTY OF EMERGENCY MEDICINE IN CHILE: 20 YEARS OF HISTORY. *Rev Médica Clínica Las Condes*. 2017;28(2):163-169. doi:10.1016/j.rmclc.2017.04.002
20. Lima MLC de, Souza ER de, Deslandes SF, Kelly A, Cabral AP de S. An analysis of prehospital care for victims of accidents and violence in Recife, Brazil. *Rev Salud Publica Bogota Colomb*. 2010;12(1):27-37.
21. Juran S, Broer PN, Klug SJ, et al. Geospatial mapping of access to timely essential surgery in sub-Saharan Africa. *BMJ Glob Health*. 2018;3(4):e000875. doi:10.1136/bmjgh-2018-000875
22. Raykar NP, Bowder AN, Liu C, et al. Geospatial mapping to estimate timely access to surgical care in nine low-income and middle-income countries. *Lancet Lond Engl*. 2015;385 Suppl 2:S16. doi:10.1016/S0140-6736(15)60811-X
23. Inter-facility transfer of surgical emergencies in a developing country: Effects on management and surgical outcomes. - PubMedâNCBI. https://www.ncbi.nlm.nih.gov/pubmed/24178181. Accessed June 25, 2019.
24. Alkire BC, Raykar NP, Shrime MG, et al. Global access to surgical care: A modelling study. *Lancet Glob Health*. 2015;3(6):e316-323. doi:10.1016/S2214-109X(15)70115-4
25. Massenburg BB, Saluja S, Jenny HE, et al. Assessing the Brazilian surgical system with six surgical indicators: A descriptive and modelling study. *BMJ Glob Health*. 2017;2(2):e000226. doi:10.1136/bmjgh-2016-000226
26. Kim J, Barreix M, Babcock C, Bills CB. Acute Care Referral Systems in Liberia: Transfer and Referral Capabilities in a Low-Income Country. *Prehospital Disaster Med*. 2017;32(6):642-650. doi:10.1017/S1049023X1700677X
27. (PDF) Impact of inter-facility transport on maternal mortality in the Free State Province | Marthinus Gerhardus Schoon. https://www.researchgate.net/publication/252323521_Impact_of_inter-facility_transport_on_maternal_mortality_in_the_Free_State_Province. Accessed June 25, 2019.
28. Giedion U, Uribe MV. Colombia's Universal Health Insurance System. *Health Aff (Millwood)*. 2009;28(3):853-863. doi:10.1377/hlthaff.28.3.853
29. OECD. *Colombia: Policy Priorities for Inclusive Development*. OECD; 2015. doi:10.1787/9789264233393-en
30. Colombia 2017 OECD economic survey overview. https://www.oecd.org/eco/surveys/Colombia-2017-OECD-economic-survey-overview.pdf. Accessed July 5, 2019.
31. Morgan R, Ensor T. The regulation of private hospitals in Asia. *Int J Health Plann Manage*. 2016;31(1):49-64. doi:10.1002/hpm.2257
32. Mills A. Health care systems in low- and middle-income countries. *N Engl J Med*. 2014;370(6):552-557. doi:10.1056/NEJMra1110897
33. Vargas I, Vázquez ML, Mogollón-Pérez AS, Unger J-P. Barriers of access to care in a

managed competition model: Lessons from Colombia. *BMC Health Serv Res*. 2010;10:297. doi:10.1186/1472-6963-10-297
